# Analysis of variable-order fractional enzyme kinetics model with time delay

**DOI:** 10.1038/s41598-025-16382-x

**Published:** 2025-10-01

**Authors:** K. Agilan, S. Naveen, S. Suganya, V. Parthiban

**Affiliations:** Department of Mathematics, School of Advanced Sciences, Vellore Institute of Technology, Chennai, Tamilnadu 600127 India

**Keywords:** Enzyme kinetics, Variable-order Caputo fractional derivative, Stability analysis, Delay differential equation, Numerical simulation, Chemistry, Mathematics and computing

## Abstract

In enzymatic reactions, studying reaction rates and mechanisms helps us understand how concentration, temperature, and catalysts influence the speed of chemical transformations. This field is critical for optimizing processes in biotechnology, pharmaceuticals, and food industries. Traditional enzyme kinetics models may overlook the influence of past system states. In this paper, we propose a variable-order Caputo fractional derivative enzyme kinetics model that incorporates constant time delays to capture memory effects and nonlocal behavior more accurately. We establish the existence and uniqueness of solutions using fixed-point theory. The proposed model stability is analyzed through Ulam–Hyers and generalized Ulam–Hyers concepts. A robust and an effective numerical approach is employed to reveal the intricate dynamics of the model and demonstrate the significance of the variable-order Caputo fractional derivative with time delay. Incorporating a delay term and employing the variable-order Caputo fractional derivative, this model refines conventional enzyme kinetics, leading to a more precise characterization of biological catalytic processes.

## Introduction

Enzyme kinetics is a fundamental component of systems biology, which aims to understand how complex biological networks function. Studying mathematical models for enzyme kinetics is crucial for understanding the fundamental principles governing biochemical reactions. Enzymes play a vital role in regulating metabolic processes, and their reaction rates are influenced by factors such as substrate concentration, enzyme availability, and environmental conditions. Traditional experimental approaches provide valuable insights, but they are often limited in capturing complex interactions and predicting system behavior under varying conditions. Recently many researchers have been investigating dynamics of fractional-order models. See^1–13^

Mathematical models offer a systematic framework to describe enzyme-substrate dynamics, estimate key parameters, and simulate reaction mechanisms with high accuracy. These models are essential for optimizing industrial and pharmaceutical applications, such as drug development, fermentation processes, and biotechnology, where precise control of enzymatic reactions is required^14^. Fractional calculus has emerged as a powerful framework to model complex biochemical processes that exhibit memory and hereditary effects. The application of fractional-order dynamics in science and technology has expanded across various fields, including physics, chemistry, biology, viscoelasticity, bioengineering, nanoparticle-substrate interactions, control theory, epidemiology, ecology, sociology, signal processing, robotics, system modeling and identification, telecommunications, electronics, finance, engineering, and other applied sciences^15–23^. These models are very useful in biological systems because they can capture intricate dynamics that are not captured by integer-order models. Long-term memory and residual effects, which are crucial to biological processes, can be well described using fractional calculus. In recent years, fractional calculus has emerged as a compelling mathematical framework for modeling complex biochemical processes that exhibit memory and hereditary effects. Unlike classical integer-order models, such as the Michaelis–Menten formulation^24^, which assume that reaction rates depend only on the present state and respond instantaneously to changes, fractional-order models incorporate the influence of past system states through non-local operators. This is particularly relevant in enzyme kinetics, where processes such as slow substrate binding and unbinding, conformational rearrangements of enzymes, and allosteric regulation introduce time-dependent behavior and history-dependent dynamics. By accounting for these effects, fractional derivatives provide a more realistic and flexible representation of enzymatic reactions, capturing features like delayed response, long-range temporal correlations, and gradual adaptation to changing biochemical environments that classical models may overlook. Recent studies have shown that the fractional order in fractional calculus models can be conceptually linked to fractal dimensions, as both characterize the complexity and irregularity of a system. In the context of enzyme kinetics, enzyme binding sites and reaction interfaces often exhibit fractal-like geometries whose irregular structures can significantly affect reaction rates. This perspective finds parallels in materials science, where, for instance, the fractal dimensions of porous concrete have been correlated with its mechanical strength. Drawing on this analogy helps to justify the use of fractional calculus in modeling enzymatic processes, as it naturally captures the influence of complex, heterogeneous structures on system dynamics and reaction kinetics. The study of variable-order fractional derivatives enhances the realism and accuracy of dynamic process modeling, resulting in superior predictive capabilities and optimized solutions across various scientific and industrial domains. Current developments have introduced numerous dynamical systems incorporating fractional variable-order derivatives, as seen in^25–29^. By extending to variable-order fractional derivatives, the model can further capture dynamic changes in memory effects over time, reflecting how enzymatic activity adapts to changing biochemical environments. This enriched framework provides a closer representation of the true kinetics of enzymatic processes compared to classical integer-order models.

In^30^ the authors are discussed the stability and bifurcation analysis of fractional-order tumor–macrophages interaction model with multi-delays. Optimal control analysis of fractional order delayed SIQR model for COVID-19 is presented in^31^. In^32^ the authors are investigated the analysis of a class of fractal hybrid fractional differential equation with application to a biological model. In^33^ dynamical behavior of a time-fractional biological model via an efficient numerical method. Modeling and optimal analysis of lung cancer cell growth and apoptosis with fractional-order dynamics is investigated in^34^. In^35^ the authors discussed the chaos in fractional-order glucose–insulin models with variable derivatives: Insights from the Laplace–adomian decomposition method and generalized Euler techniques. In^36^ on variable-order Salmonella bacterial infection mathematical model is presented. A new numerical strategy for solving nonlinear singular Emden-Fowler delay differential models with variable order is studied in^37^. In^38^ the authors are discussed the variable order fractional diabetes models with numerical treatment. The optimal control problem of hybrid fracInt variable-order mathematical model for Covid-19 with time delay is presented in^39^. Additionally the fractional and variable order derivative systems are applied in the various fields: application of Chen system^40^, finite-time fuzzy synchronization of chaotic systems^41^, adaptive fuzzy control for practical fixed-time synchronization of fractional-order chaotic system^42^, reversible two-step enzymatic reaction with time fractional derivative^43^.

Various mathematical models have been introduced to explore the complex regulation of enzyme kinetics, shedding light on key factors that influence enzymatic reactions and their efficiency. For instance, Khan et al.^44^ proposed a fractional-order model that provides multiple solutions compared to classical models, suggesting increased complexity and a potentially more comprehensive representation of enzyme kinetics. In^45^, the authors applied hybrid proportional fractional derivatives, namely the constant proportional Caputo-Fabrizio (CPCF) and constant proportional Atangana-Baleanu-Caputo (CPABC) operators, to enzyme kinetics, demonstrating improved forecasting and dynamic modeling. Furthermore, various numerical methods have been developed to model enzyme kinetics by incorporating different fractional-order derivatives, which have been extensively studied. Moreover, the Ulam-Hyers stability analysis are applied in different scenarios: Applications of RLC circuit system^46^, $\psi$-Hilfer abstract fractional functional differential equation^47^, nonlinear fractional reaction–diffusion equations with delay^48^, neutral stochastic functional differential equations^49^. For instance, Ahmad et al^50^ performed a comparative analysis of cooperative chemical reactions using both singular and nonsingular kernels, revealing how kernel choice affects reaction speed and system memory. Chethan et al.^51^ proposed a high-performance computational approach to study a reversible two-step enzymatic reaction described by time-fractional derivatives, demonstrating improved simulation accuracy. Sabarinathan et al.^52^ explored the stability of enzyme kinetics through a fractal-fractional framework, offering new mathematical insights into reaction dynamics. Additionally, Radhakrishnan et al.^53^ analyzed a nonlinear fractional-order biochemical reaction model, supporting their findings with detailed numerical simulations. Collectively, these works underscore the growing importance and versatility of fractional and fractal-fractional modeling approaches in biochemical kinetics, motivating our present study. Furthermore, the various numerical approximations are applied for fractional order and variable order differential equations such as, in^54^, the variable order Adams-Bashforth-Milton methodology for Lotka-Volterra predator prey system, Runge-Kutta 4th order and Modified Euler method for Caputo derivative of LC and RC circuits system^55^. In^56^, fractional derivative with non-local and non-singular kernel: application to chaotic model. In^57^, Two dimensional fractional Euler polynomial method for fractional diffusion-wave equation in^58^. In^59^ the application of Newton’s polynomial interpolation scheme for power-law kernel.

### The proposed work’s motivations and contributions

In this study, we adopt the Caputo definition of the variable-order fractional derivative for modeling enzyme kinetics. This choice is motivated by its important physical advantage: the Caputo derivative allows the use of standard initial conditions expressed in terms of integer-order derivatives, such as experimentally measurable initial concentrations of substrate and enzyme. In contrast, other commonly used definitions like the Riemann–Liouville derivative require initial conditions involving fractional integrals, which lack direct physical or biological interpretation. Although the Atangana–Baleanu derivative offers benefits for modeling non-singular kernel processes, our focus is specifically on enzyme systems characterized by classical power-law memory effects, for which the Caputo approach is particularly suitable and widely accepted. This ensures that the model remains both mathematically rigorous and biologically meaningful, closely aligning with experimental observations in biochemical kinetics. Enzymatic reactions are often influenced by complex environmental and cellular factors such as temperature fluctuations, pH variations, or substrate/enzyme concentration changes, which naturally introduce time-varying memory effects. The variable-order fractional derivative allows the model to reflect that the “memory strength” (the influence of past states) is not constant but can evolve over time, capturing phenomena like enzyme saturation, inhibition, or activation phases. This approach acknowledges that enzyme systems might exhibit adaptation or fatigue, making the reaction kinetics history-dependent in a non-uniform way. The proposed variable-order fractional model has practical relevance in several biological and industrial contexts. For instance, in pharmaceutical manufacturing, enzyme-catalyzed reactions often experience variations in activity due to fluctuating temperature or pH levels over time. Similarly, in food technology processes like fermentation, enzyme efficiency changes gradually as substrates deplete and products accumulate. Classical integer-order models, which assume memoryless kinetics, may not capture these subtle time-dependent behaviors. By contrast, the variable-order fractional approach accounts for evolving memory effects, providing a more accurate and flexible description of reaction dynamics. This can lead to improved predictions of process outcomes and better control strategies in industrial applications.

Recent developments have demonstrated how useful delay fractional differential equations (DFDEs) can be in simulating dynamics in the real world^60–64^. These mathematical frameworks propose that the evolution of a function at a given time is governed by its prior states. By incorporating time delays, the model accounts for biochemical reaction steps that do not occur instantaneously, such as conformational changes in enzymes or intermediate complex formation. Allosteric enzymes like phosphofructokinase demonstrate time lags through cooperative binding mechanisms, multi-enzyme complexes such as pyruvate dehydrogenase exhibit delays during substrate channeling, and cofactor-dependent systems show lags during regeneration cycles. Additionally, inducible enzyme systems and processive enzymes display characteristic time delays that are physiologically significant^65,66^. Due to time delays, the intricate interactions between different reaction stages and regulatory mechanisms in enzyme kinetics can cause oscillatory behavior in solutions. Understanding these oscillations is essential for optimizing enzymatic processes and improving their applications in biotechnology and pharmaceutical industries. Even fixed-order fractional models, though capable of representing long-term memory, are limited by the assumption of a constant memory strength throughout the reaction. Moreover, many existing models neglect biologically realistic time delays that arise from intermediate complex formation or conformational changes.Traditional constant-order fractional models assume that the memory effect quantified by a fixed fractional order $\alpha$ remains unchanged over the course of the reaction. While these models have been effective in capturing long-term memory and non-local behavior, they may oversimplify biological systems where the memory effect itself can evolve over time. In contrast, our variable-order approach, where the order $\delta (t)$ is allowed to vary as a smooth function of time, reflects adaptive processes such as substrate depletion, enzyme activation or inhibition, and conformational changes. This additional flexibility enables the model to more accurately describe complex kinetic behaviors, like transient dynamics or delayed product accumulation, which are often observed experimentally. Consequently, the variable-order model not only generalizes the constant-order case but also offers enhanced predictive power and biological interpretability. Motivated by the above discussion, we propose a delay model with a variable-order Caputo fractional derivative for enzyme kinetics, which characterizes the system’s variable memory and allows us to capture both adaptive memory effects and intrinsic time-lag behaviors observed in real enzymatic processes. To the best of our knowledge, this model remains unexplored in the existing literature. Thus, the mathematical findings presented here are both innovative and significant.

The key contributions of this study are as follows:

- Developed a novel variable-order Caputo fractional derivative (VOCFD) enzyme kinetics model with constant time delays, capturing adaptive memory effects and biologically realistic time-lag behavior.
- Performed a comprehensive qualitative analysis, demonstrating positivity, boundedness, and proving the existence and uniqueness of solutions using fixed-point theorems.
- Conducted a detailed stability analysis of the model within the Ulam–Hyers and generalized Ulam–Hyers frameworks, ensuring the reliability of the solution under perturbations.
- Designed and implemented a new, robust numerical method to simulate the proposed model and illustrate the effects of variable-order memory and delays on enzymatic reaction dynamics.

**Figure 1:**
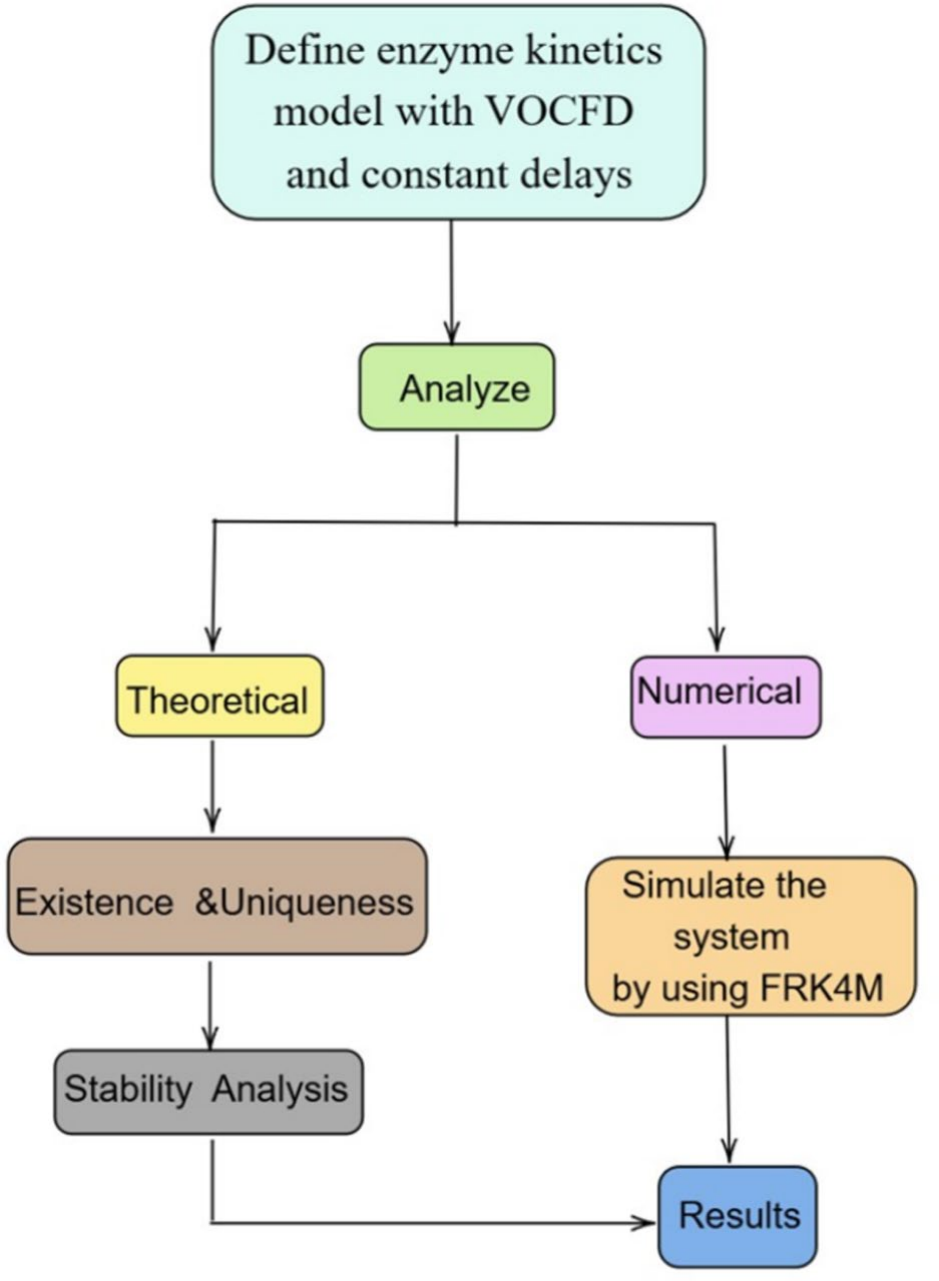
Schematic workflow of the proposed study.

To enhance clarity, Fig. 1 presents a complete workflow diagram diagram summarizing the proposed methodology. The diagram outlines the main components of this study, including the formulation of the enzyme kinetics model with variable-order Caputo fractional derivative and time delays, the qualitative analysis ensuring well-posedness, the stability analysis within the Ulam–Hyers framework, and the implementation of a numerical method to simulate and analyze the model dynamic behavior.

### Structure of the paper

The structure of this paper is as follows: A VOCFD model for enzyme kinetics with delay is presented in "A mathematical model for the enzyme kinetics" section. In "Preliminaries" section, basic definitions have been discussed. In Fundamental properties" section, positivity and boundness, and in "Existence and uniqueness of solution" section, existence and uniqueness results of the system are determined; the stability analysis is discussed in "Stability analysis of variable-order enzyme kinetics model" section. In "Numerical simulation" section, discussions and simulations using numerical approaches are provided. The study is summarized in the conclusion given in "Conclusion" section.

## A mathematical model for the enzyme kinetics

To examine the mathematical model in order to use the previously described research data to look into the presence of solutions in an enzyme kinetics model^67^. The proposed fractional-order enzyme kinetics model provides several advantages over the classical integer-order model. In real biochemical systems, reactions exhibit memory and hereditary properties because the present state depends not only on the current concentrations but also on the past history of the system. The fractional-order derivative effectively captures this memory effect, whereas the integer-order model assumes an instantaneous response and ignores past dynamics. Furthermore, the fractional-order framework offers greater flexibility in describing complex kinetic behaviors, including anomalous diffusion and subdiffusion commonly observed in biochemical processes. By incorporating variable order, the model adapts to time-dependent dynamics and better reflects the biological reality compared to the rigid structure of integer-order models. Furthermore, the existence of a solution means that the enzyme kinetics process described by model (2) is mathematically and biologically feasible; that is, for given initial concentrations of substrate, enzyme, and product, the system will evolve in a well-defined way over time. Uniqueness ensures that the reaction follows a single predictable pathway under the same initial and parameter conditions, avoiding multiple or conflicting behaviors for the same situation, which aligns with the deterministic nature of biochemical reactions. The boundedness of the solution implies that the concentrations of substrate, enzyme, complex, and product remain finite and biologically realistic over time, avoiding unphysical scenarios such as negative or infinitely large concentrations. Together, these properties guarantee that the proposed fractional-order model reflects a stable and meaningful biological process.

The given conditions in the proposed system are independent of one another and satisfy $$\mathbb {N}({t})=$$$$\mathbb {S}({t})$$$$+\mathbb {E}({t})+$$$$+\mathbb {H}({t})+$$$$\mathbb {P}({t})$$. The $$\mathbb {N}$$ value represents the terms presented in the relevant system of reactions. The concentration of a substance is represented by square brackets [ ] and $$[\mathbb {S}]=\mathbb {S},[\mathbb {E}]=\mathbb {E}$$, and $$[\mathbb {H}]=\mathbb {H}$$ as follows:

1 $$\begin{aligned} {\left\{ \begin{array}{ll} \frac{d \mathbb {S}}{d {t}}={\beta }_{2} \mathbb {H}-{\beta }_{1} \mathbb {E}, \\ \frac{d \mathbb {E}}{d {t}}={\beta }_{3} \mathbb {H}+{\beta }_{2} \mathbb {H}-{\beta }_{1} \mathbb {E S}, \\ \frac{d \mathbb {H}}{d {t}}=-{\beta }_{3} \mathbb {H}-{\beta }_{2} \mathbb {H}+{\beta }_{1} \mathbb {E}(t-\tau _{1}) \mathbb {S}(t-\tau _{2}), \\ \frac{d \mathbb {P}}{d {t}}={\beta }_{3} \mathbb {P} .\end{array}\right. } \end{aligned}$$

Examine the variable-order Caputo fractional derivative in the enzyme kinetics and study existence, uniqueness, and numerical simulations into account. Here is a summary of our methodology:

2 $$\begin{aligned} {\left\{ \begin{array}{ll} \mathscr {D}^{\delta (t)} \mathbb {S} (t)={\beta }_{2} \mathbb {H}-{\beta }_{1} \mathbb {E S}, \\ \mathscr {D}^{\delta (t)} \mathbb {E}(t)={\beta }_{3} \mathbb {H}+{\beta }_{2} \mathbb {H}-{\beta }_{1} \mathbb {E S}, \\ \mathscr {D}^{\delta (t)} \mathbb {H}(t)=-{\beta }_{3} \mathbb {H}-{\beta }_{2} \mathbb {H}+{\beta }_{1} \mathbb {E}(t-\tau _{1}) \mathbb {S}(t-\tau _{2}), \\ \mathscr {D}^{\delta (t)}\mathbb {P}(t)={\beta }_{3} \mathbb {P}. \end{array}\right. } \end{aligned}$$

With the initial condition, the enzyme kinetics model’s impact becomes $$\mathbb {S}(0)=\mathbb {S}_{0},$$$$\mathbb {E}(0)=\mathbb {E}_{0},$$$$\mathbb {H}(0)=\mathbb {H}_{0},$$$$\mathbb {P}(0)=\mathbb {P}_{0}$$. Here, it $$\mathscr {D}^{\delta (t)}$$ stands for the variable order Caputo fractional derivative for $$\delta (t) \in (0,1]$$.

In this work, the fractional order $\delta (t)$ is defined as a smooth, time-dependent function reflecting gradual biological changes, such as temperature shifts or product inhibition, that affect system memory. Biologically, $\delta (t)$ quantifies how strongly past states influence current reaction rates. Its variation can be inferred from experimental observations like slow relaxation or delayed responses in enzyme activity, thus linking the abstract mathematical concept of variable-order derivatives to measurable biochemical behavior. When one molecule of enzyme $\mathbb {E}$ is combined with one molecule of substrate $\mathbb {S}$, an enzyme substrate $\mathbb {H}$ composed of one molecule and the product $\mathbb {P}$ is created, as can be seen when examining the chemical reaction (2). Here, ${\beta }_{1}$ the rate at which enzymes develop ${\beta }_{2}$ denotes the rate at which products are created and ${\beta }_{3}$ denotes catalysis. We introduce two constant delays, $\tau _{1}$ and $\tau _{2}$, to represent fixed time-lags associated with intermediate steps in the enzymatic reaction process, such as conformational changes or complex formation. These constant delays simplify numerical implementation within the Fractional Runge Kutta method of the fourth order (FRK4M) framework while effectively capturing biologically relevant time-lag effects. A detailed description of the model parameters is provided in Table 1.

**Table 1:** Description of the model parameters.

Symbol	Meaning
$\mathbb {S}(t)$	Substrate concentration at time $t$
$\mathbb {E}(t)$	Free enzyme concentration at time $t$
$\mathbb {H}(t)$	Enzyme-substrate complex concentration at time $t$
$\mathbb {P}(t)$	Product concentration at time $t$
$\beta _{1}$	Rate constant for enzyme binding
$\beta _{2}$	Rate constant for product formation
$\beta _{3}$	Rate constant for catalysis
$\tau _{1}, \tau _{2}$	Constant delays

## Preliminaries

In this section, we discuss basic concepts of the variable order Caputo fractional derivative and provide definitions that are necessary to obtain the main results of this study. Moreover, in this study, we consider the Banach space denoted as $\{G$ : $G({t}) \in C([0,1]) \rightarrow R\}$ under the norm $\Vert G\Vert =\max _{{t} \in [0,1]}|G({t})|$.

### Definition 3.1

^55^Regarding the same function *G* as discussed earlier, the RL integral of order $0<\delta (t) \le 1$, can be defined as follows:

$$\begin{aligned} \mathscr {I}^{\delta (t)} G({t})=&\frac{1}{\Gamma (\delta (t)} \int _{0}^{t} G(v)({t}-v)^{\delta (t)-1} d v. \\ \end{aligned}$$

By using a successive iterative technique, the variable order Caputo fractional model is used (2). To accomplish this, we apply Definition 3.1’s integral to the suggested model (2).

3 $$\left\{ {\begin{array}{*{20}l} {{\mathbb{S}}(t) = \;{\mathbb{S}}(0) + \frac{1}{{\Gamma (\delta (t))}}\int_{0}^{t} {(t - v)^{{\delta (t) - 1}} } \left[ {\beta _{2} {\mathbb{H}} - \beta _{1} {\mathbb{E}\mathbb{S}}} \right]dv} \hfill \\ {{\mathbb{E}}(t) = \;{\mathbb{E}}(0) + \frac{1}{{\Gamma (\delta (t))}}\int_{0}^{t} {(t - v)^{{\delta (t) - 1}} } \left[ {\beta _{3} {\mathbb{H}} + \beta _{2} {\mathbb{H}} - \beta _{1} {\mathbb{E}\mathbb{S}}} \right]dv} \hfill \\ {{\mathbb{H}}(t) = \;{\mathbb{H}}(0) + \frac{1}{{\Gamma (\delta (t))}}\int_{0}^{t} {(t - v)^{{\delta (t) - 1}} } \left[ { - \beta _{3} {\mathbb{H}} - \beta _{2} {\mathbb{H}} + \beta _{1} {\mathbb{E}}(t - \tau _{1} ){\mathbb{S}}(t - \tau _{2} )} \right]dv} \hfill \\ {{\mathbb{P}}(t) = \;{\mathbb{P}}(0) + \frac{1}{{\Gamma (\delta (t))}}\int_{0}^{t} {(t - v)^{{\delta (t) - 1}} } \left[ {\beta _{3} P} \right]dv.} \hfill \\ \end{array} } \right.$$

Setting $\mathscr {L}_{j}$ for $j=1,2,3,4$, then we have

4 $$\begin{aligned} {\left\{ \begin{array}{l} \mathscr {L}_{1}({t}, \mathbb {S}) ={\beta }_{2} \mathbb {H}-{\beta }_{1} \mathbb {E S} \\ \mathscr {L}_{2}({t}, \mathbb {E}) ={\beta }_{3} \mathbb {H}+{\beta }_{2} \mathbb {H}-{\beta }_{1} \mathbb {E S} \\ \mathscr {L}_{3}({t}, \mathbb {H}) =-{\beta }_{3} \mathbb {H}-{\beta }_{2} \mathbb {H}+{\beta }_{1} \mathbb {E}(t-\tau _{1}) \mathbb {S}(t-\tau _{2}) \\ \mathscr {L}_{4}({t}, \mathbb {P}) ={\beta }_{3} \mathbb {P}. \end{array}\right. } \end{aligned}$$

## Fundamental properties

Here, we demonstrate that the model (2) has the boundedness and positivity.

### Positivity of the model

#### Theorem 4.1

*The solution to the model (*2*) has the positivity property*.

#### Proof

From model (2), we have

5 $$\begin{aligned} {\left\{ \begin{array}{ll}\left. \mathbb {S}({t})\right| _{\mathbb {S}=0} & ={\beta }_{2} \mathbb {H}-{\beta }_{1} \mathbb {E} \mathbb {S}>0, \\ \left. \mathbb {E}({t})\right| _{\mathbb {E}=0} & ={\beta }_{3} \mathbb {H}+{\beta }_{2} \mathbb {H}-{\beta }_{1} \mathbb {E S}>0, \\ \left. \mathbb {H}({t})\right| _{\mathbb {H}=0} & =-{\beta }_{3} \mathbb {H}-{\beta }_{2} \mathbb {H}+{\beta }_{1} \mathbb {E}(t-\tau _{1}) \mathbb {S}(t-\tau _{2})>0, \\ \left. \mathbb {P}({t})\right| _{\mathbb {P}=0} & ={\beta }_{3} \mathbb {P} .\end{array}\right. } \end{aligned}$$

The outcome shows that none of the model’s parameters or reactions are negative. The proof can be effectively concluded since all solutions for our model (2) are guaranteed to be non-negative.$\square$

### Boundedness of the model

#### Theorem 4.2

*The solution of our model (* 2 *), denoted as*

$Y=\{\mathbb {S}({t}), \mathbb {E}({t}),$ $\left. \mathbb {H} ({t}), \mathbb {P}({t}) \in \mathbb {R}^{4}: 0<\mathbb {N} \le \frac{\varphi }{\rho }\right\}$*, adheres to this invariant. The solution remains bounded within the positive invariant region* $\mathbb {R}^{4}$ *while considering initial conditions of* $\mathbb {S}>0, \mathbb {E}>0, \mathbb {H} \ge 0$*, and* $\mathbb {P} \ge 0$.

#### Proof

Assume that $\mathbb {N}({t})=\mathbb {S}({t})+\mathbb {E}({t})+\mathbb {H}({t})+{P}({t})$ and $\hat{\mathbb {N}}({t})=$ $\mathbb {S}({t})+\mathbb {E}({t})+\mathbb {H} \hat{( } {t})+\hat{\mathbb {P}}({t})$ sum of the model (2), we get

6 $$\begin{aligned} \widehat{\mathbb {N}}({t})=\varphi -\rho \mathbb {N}, \end{aligned}$$

then integrating Eq. 6, we obtain $Y=\left\{ \mathbb {S}({t})+\mathbb {E}({t})+\mathbb {H}({t})+\mathbb {P}({t}) \le \frac{\varphi }{\rho }\right\}$.

When *t* becomes very large i.e., ${t} \rightarrow \infty$, it implies that $\frac{\varphi }{\rho }$ is the supremum of $\mathbb {N}$, making it a positive invariant for the model (2).$\square$

## Existence and uniqueness of solution

In this context, we employ a fixed-point approach to examine the existence and uniqueness of a solution. To simplify the analysis, under assumption $$(\mathscr {H})$$ : (H), we take into account the following considerations. For the $$\mathbb {S}({t}), \hat{\mathbb {S}}({t}), \mathbb {E}, \hat{\mathbb {E}}({t}), \mathbb {H}({t}), \hat{\mathbb {H}}({t}), \mathbb {P}({t})$$ and $$\hat{\mathbb {P}}({t}) \in \textrm{L}[0,1]$$ be continuous function, such that $$\Vert \mathbb {S}$$$$\Vert \le b_{1},$$$$\Vert \mathbb {E}$$$$\Vert \le b_{2}$$, $$\Vert \mathbb {H}\Vert \le b_{3},$$$$\Vert \mathbb {P}\Vert \le b_{4}$$ for non-negative constant $$b_{1}, b_{2}, b_{3}, b_{4}>0$$. Additionally, we introduce the following constants $$\Phi _{1}={\beta }_{1} b_{2}, \Phi _{2}={\beta }_{1} b_{1}, \Phi _{3}=$$ $${\beta }_{3}+{\beta }_{2}, \Phi _{4}={\beta }_{3}$$.

### Theorem 5.1

*The kernels* $\mathscr {L}_{j},$ *for* $j=1,2,3,4$ *holds on Lipschitz condition, if the assumption* $(\mathscr {H})$ *is satisfied and* $\Phi j<1$ *for* $j=1,2,3,4.$

### Proof

The desired outcome is achieved under the assumption $(\mathscr {H})$. Similarly, by applying analogous reasoning to $\mathscr {L}_{1}({t}, \mathbb {S})$(t,S) and utilizing the Lipschitz condition, we derive the following result.

$$\begin{aligned} \left\| \mathscr {L}_{1}(\mathbb {S})-\mathscr {L}_{1}(\hat{\mathbb {S}})\right\| =&\left\| \left[ {\beta }_{2} \mathbb {H}-{\beta }_{1} \mathbb {E} \mathbb {S}\right] -\left[ {\beta }_{2} \mathbb {H}-{\beta }_{1} \mathbb {E} \hat{\mathbb {S}}\right] \right\| ,\\ \le&{\beta }_{1}\Vert \mathbb {S}-\hat{\mathbb {S}}\Vert \Vert \mathbb {E}\Vert =\Phi _{1}\Vert \mathbb {S}-\hat{\mathbb {S}} \Vert \end{aligned}$$

this shows that $\Phi _{1}={\beta }_{1} b_{2}$.

Consequently, $\mathscr {L}_{1}$ satisfies the Lipschitz condition with a Lipschitz constant $\Phi _{1}$. Similarly, the other kernels also adhere to the Lipschitz condition.

$$\begin{aligned} \left\| \mathscr {L}_{2}(\mathbb {E})-\mathscr {L}_{2}(\hat{\mathbb {E}})\right\| =&\left\| \left[ {\beta }_{3} \mathbb {H}+{\beta }_{2} \mathbb {H}-{\beta }_{1} \mathbb {E} \mathbb {S}\right] -\left[ {\beta }_{3} \mathbb {H}+{\beta }_{2} \mathbb {H}-{\beta }_{1} \hat{\mathbb {E}} \mathbb {S}\right] \right\| ,\\ \le&{\beta }_{1}\Vert \mathbb {E}-\hat{\mathbb {E}}\Vert \Vert \mathbb {S}\Vert =\Phi _{2} \Vert \mathbb {E}-\hat{\mathbb {E}} \mid , \end{aligned}$$

where $\Phi _{2}={\beta }_{1} b_{1}$. Thus, $\mathscr {L}_{2}$ fulfiles the Lipschitz condition with constant $\Phi _{2}$. Then

$$\begin{aligned} \left\| \mathscr {L}_{3}(\mathbb {H})-\mathscr {L}_{3}(\hat{\mathbb {H}})\right\| =&\left\| \left[ -{\beta }_{3} \mathbb {H}-{\beta }_{2} \mathbb {H}+{\beta }_{1} \mathbb {E S}\right] -\left[ -{\beta }_{3} \hat{H}-{\beta }_{2} \hat{H}+{\beta }_{1} \mathbb {E S}\right] \right\| ,\\ \le&\left( {\beta }_{3}+{\beta }_{2}\right) \Vert \mathbb {H}-\hat{\mathbb {H}}\Vert =\Phi _{3} \Vert \mathbb {H}-\hat{\mathbb {H}} \Vert , \end{aligned}$$

where $\Phi _{3}={\beta }_{3}+{\beta }_{2}$. Thus, $\mathscr {L}_{3}$ fulfiles the Lipschitz condition with constant $\Phi _{3}$. Then

$$\begin{aligned} \left\| \mathscr {L}_{4}(\mathbb {P})-\mathscr {L}_{4}(\hat{\mathbb {P}})\right\| =\left\| \left[ {\beta }_{3} \mathbb {P}\right] -\left[ {\beta }_{3} \hat{\mathbb {P}}\right] \right\| , \le {\beta }_{3}\Vert \mathbb {P}-\hat{\mathbb {P}}\Vert =\Phi _{4} \Vert \mathbb {P}-\hat{\mathbb {P}}|\Vert ., \end{aligned}$$

Where $\Phi _{4}={\beta }_{3}$, it follows that $\mathscr {L}_{3}$ satisfies the Lipschitz condition with the constant $\Phi _{4}$. Consequently, from Eq. (3), all kernels $\mathscr {L}_{j}$, for $j=1,2,3,4$, satisfy the Lipschitz property, leading to the desired result.

$$\begin{aligned} {\left\{ \begin{array}{l} \mathbb {S}({t})= \mathbb {S}(0)+\frac{1}{\Gamma \left( {\delta (t)}\right) } \int _{0}^{{t}}({t}-v)^{{\delta (t)}-1} \mathscr {L}_{1}(v, \mathbb {S}(v)) d v\\ \mathbb {E}({t})= \mathbb {E}(0)+\frac{1}{ \Gamma ({\delta (t)})} \int _{0}^{t}({t}-v)^{{\delta (t)}-1} \mathscr {L}_{2}(v, \mathbb {E}(v)) d v\\ \mathbb {H}({t})= \mathbb {H}(0)+\frac{1}{ \Gamma ({\delta (t)})} \int _{0}^{t}({t}-v)^{{\delta (t)}-1} \mathscr {L}_{3}(v, \mathbb {H}(v)) d v\\ \mathbb {P}({t})= \mathbb {P}(0)+\frac{1}{\Gamma ({\delta (t)})} \int _{0}^{t}({t}-v)^{{\delta (t)}-1} \mathscr {L}_{4}(v, \mathbb {P}(v)) d v. \end{array}\right. } \end{aligned}$$

$$\square$$

### Theorem 5.2

*If the variable-order Caputo fractional model (*2*) has a solution under assumption (* $\hat{H}$ *), then*

$$\zeta =\max \left[ \Phi _{1}, \Phi _{2}, \Phi _{3}, \Phi _{4}\right] <1.$$

### Proof

We define four functions $\omega _{1 \kappa }, \omega _{2 \kappa }, \omega _{3 \kappa }$, and $\omega _{4 \kappa }$ using a sequential iterative approach based on (2), as follows.

7 $$\begin{aligned} {\left\{ \begin{array}{ll} \omega _{1 \kappa }({t})=\mathbb {S}_{\kappa +1}({t})-\mathbb {S}({t}), \omega _{2 \kappa }({t})=\mathbb {E}_{\kappa +1}({t})-\mathbb {E}({t}), \\ \omega _{3 \kappa }({t})=\mathbb {H}_{\kappa +1}({t})-\mathbb {H}({t}), \omega _{4 \kappa }({t})=\mathbb {P}_{\kappa +1}({t})-\mathbb {P}({t}). \end{array}\right. } \end{aligned}$$

As a result, we obtain the following.

8 $$\begin{aligned} {\left\{ \begin{array}{ll} \left\| \omega _{1 \kappa }({t})\right\| & = \frac{1}{ \Gamma ({\delta (t)})} \int _{0}^{{t}}({t}-v)^{{\delta (t)}-1}\left\| \left[ \mathscr {L}_{1}\left( v, \mathbb {S}_{\kappa }(v)\right) -\mathscr {L}_{1}\left( v, \mathbb {S}_{\kappa }(v)\right) \right] \right\| d v \\ & \le { \frac{{{t}}^{{\delta (t)}}}{ \Gamma (\delta (t)+1)} \Phi _{1}\left\| \mathbb {S}_{\kappa }-\mathbb {S}\right\| } \\ & \le {\frac{{{t}}^{\delta (t)}}{ \Gamma (\delta (t))+1)} \Phi _{1}^{\kappa }\left\| \mathbb {S}_{1}-\mathbb {S}\right\| }. \end{array}\right. } \end{aligned}$$

According that, we can estimate

9 $$\begin{aligned} {\left\{ \begin{array}{ll} \left\| \omega _{2 \kappa }({t})\right\| & = \frac{1}{ \Gamma (\delta (t))} \int _{0}^{t}({t}-v)^{{\delta (t)}-1}\left\| \left[ \mathscr {L}_{2}\left( v, \mathbb {E}_{\kappa }(v)\right) -\mathscr {L}_{2}\left( v, \mathbb {E}_{\kappa }(v)\right) \right] \right\| d v \\ & \le {\frac{{{t}}^{{\delta (t)}}}{ \Gamma (\delta (t)+1)} \Phi _{2}\left\| \mathbb {E}_{\kappa }-\mathbb {E}\right\| } \\ & \le \big [{\frac{{{t}}^{\delta (t)}}{ \Gamma (\delta (t)+1)}\big ]^{\kappa } \Phi _{2}^{\kappa }\left\| \mathbb {E}_{1}-\mathbb {E}\right\| }. \end{array}\right. } \end{aligned}$$

10 $$\begin{aligned} {\left\{ \begin{array}{ll} \left\| \omega _{3 \kappa }({t})\right\| & = \frac{1}{\Gamma (\delta (t))} \int _{0}^{t}({t}-v)^{\delta (t)-1}\left\| \left[ \mathscr {L}_{3}\left( v, \mathbb {H}_{\kappa }(v)\right) -\mathscr {L}_{3}\left( v, \mathbb {H}_{\kappa }(v)\right) \right] \right\| d v \\ & \le {\frac{{{t}}^{\delta (t)}}{ \Gamma (\delta (t)+1)} \Phi _{3}\left\| \mathbb {H}_{\kappa }-\mathbb {H}\right\| } \\ & \le \big [{\frac{{{t}}^{\delta (t)}}{ \Gamma (\delta (t)+1)}\big ]^{\kappa } \Phi _{3}^{\kappa }\left\| \mathbb {H}_{1}-\mathbb {H}\right\| }. \end{array}\right. } \end{aligned}$$

Finally, we obtain

11 $$\begin{aligned} {\left\{ \begin{array}{ll} \left\| \omega _{4 \kappa }({t})\right\| & = \frac{1}{ \Gamma (\delta (t))} \int _{0}^{t}({t}-v)^{\delta (t)-1}\left\| \left[ \mathscr {L}_{4}\left( v, \mathbb {P}_{\kappa }(v)\right) -\mathscr {L}_{4}\left( v, \mathbb {P}_{\kappa }(v)\right) \right] \right\| d v \\ & \le {\frac{{{t}}^{\delta (t)}}{ \Gamma (\delta (t)+1)} \Phi _{4}\left\| \mathbb {P}_{\kappa }-\mathbb {P}\right\| } \\ & \le \big [{\frac{{{t}}^{\delta (t)}}{ \Gamma (\delta (t)+1)} \big ]^{\kappa }\Phi _{4}^{\kappa }\left\| \mathbb {P}_{1}-\mathbb {P}\right\| }. \end{array}\right. } \end{aligned}$$

Using Eqs. (8)-(11) and taking the limit on both sides as $\kappa \rightarrow \infty$, the aforementioned functions demonstrate the following property of $\omega _{j_{\kappa }}({t}) \rightarrow 0$ for $j=1,2,3,4$, given that $\Phi _{j}<1,(j=1,2,3,4)$. Thus, we establish that the model (2) has a solution, thereby completing the proof.$\square$

### Theorem 5.3

*The variable-order Caputo fractional model (*2*) possesses a unique solution provided that assumption (* $\mathscr {H})$ *holds and* ${\frac{{{t}}^{\delta (t)}}{ \Gamma (\delta (t)+1)} } \Phi _{j} \le 1,$ *for* $j \in \mathscr {N}_{1}^{4}.$

### Proof

Let us consider an alternative existing solution $(\hat{\mathbb {S}}, \hat{\mathbb {E}}, \hat{\mathbb {H}}, \hat{\mathbb {P}})$ with initial values $(\hat{\mathbb {S}}(0), \hat{\mathbb {E}}(0), \hat{\mathbb {H}}(0), \hat{\mathbb {P}}(0))$, we have

$$\begin{aligned} \hat{\mathbb {S}}({t})=&\hat{\mathbb {S}}(0)+\frac{1}{\Gamma (\delta (t))} \int _{0}^{t}({t}-v)^{\delta (t)-1} \mathscr {L}_{1}(v, \hat{\mathbb {S}}(v)) d v \end{aligned}$$

then, following:

$$\begin{aligned} \hat{\mathbb {E}}({t})=&\hat{\mathbb {E}}(0)+\frac{1}{\Gamma (\delta (t))} \int _{0}^{{t}}({t}-v)^{\delta (t)-1} \mathscr {L}_{2}(v, \hat{\mathbb {E}}(v)) d v \\ \hat{\mathbb {H}}({t})=&\hat{\mathbb {H}}(0)+\frac{1}{\Gamma (\delta (t))} \int _{0}^{t}({t}-v)^{\delta (t)-1} \mathscr {L}_{3}(v, \hat{\mathbb {H}}(v)) d v \\ \hat{\mathbb {P}}({t})=&\hat{\mathbb {P}}(0)+\frac{1}{\Gamma (\delta (t))} \int _{0}^{t}({t}-v)^{\delta (t)-1} \mathscr {L}_{4}(v, \hat{\mathbb {P}}(v)) dv. \end{aligned}$$

Again we have,

$$\begin{aligned} |\mathbb {S}-\hat{\mathbb {S}}|=\,&\frac{1}{\Gamma (\delta (t))} \int _{0}^{t}(s-v)^{\delta (t)-1}\left\| \mathscr {L}_{1}(v, \hat{\mathbb {E}}(v))-\mathscr {L}_{1}(v, \mathbb {E}(v))\right\| d v \\ \le&\frac{1}{\Gamma (\delta (t))} \int _{0}^{t}(s-v)^{\delta (t)-1} \Phi _{1}\left\| \mathbb {S}_{1}-\hat{\mathbb {S}}\right\| \\ \le&{\frac{s^{\delta (t)}}{\Gamma (\delta (t)+1)} \Phi _{1}\left\| \mathbb {S}_{1}-\hat{\mathbb {S}}\right\| }. \end{aligned}$$

Then, we have

12 $$\begin{aligned} \frac{s^{\delta (t)}}{\Gamma (\delta (t)+1)} \Phi _{1}\Vert \mathbb {S}_{1}-\hat{\mathbb {S}}\Vert \le 0. \end{aligned}$$

The inequality (12) mentioned above holds true in the case where $\Vert \mathbb {S}-$ $\hat{\mathbb {S}} \Vert =0$. This subsequently leads to the conclusion that $\mathbb {S}=\hat{\mathbb {S}}$, thereby establishing the uniqueness of the solution. The same results also exist for $\mathbb {E}, \mathbb {H}$, and $\mathbb {P}$. Thus, it can be concluded that a unique solution is admitted by the model (2).$\square$

##  Stability analysis of variable-order enzyme kinetics model

To strengthen the clarity of the stability analysis, we now provide a detailed explanation of the application of the Hyers-Ulam and generalized Hyers-Ulam stability concepts to the proposed variable-order enzyme kinetics model. Following the framework outlined in^68–71^, we establish conditions under which the approximate solution remains close to the exact solution, despite small perturbations. This analysis demonstrates that the model exhibits robustness against minor modeling errors or parameter uncertainties, which is critical for reliable simulation and interpretation of enzymatic dynamics. The inclusion of these references also situates our analysis within the broader context of stability studies for fractional and variable-order systems.The primary focus of this paper is the Hyers-Ulam stability analysis of the model (2). If and only if there is a continuous function $\vartheta _{1}$ (depending on $\mathbb {S}$ ), then the function S is a solution (13).

The novelty of this work lies in extending the Hyers–Ulam stability analysis to a variable-order Caputo fractional derivative model of enzyme kinetics, which has not been widely studied in the existing literature. Unlike most previous works that consider a constant fractional order, our model allows $\delta (t)\in (0,1]$, introducing a time-dependent memory effect and making the analysis applicable to more realistic, non-stationary biochemical processes. Additionally, we incorporate time delays in the interaction terms, which significantly increases the complexity and requires modifying the stability framework compared to models without delays. Another important distinction is that our study combines existence, uniqueness, boundedness, and Hyers–Ulam stability within the same theoretical setting, ensuring a comprehensive understanding of the system’s behavior. Finally, we support the theoretical results with numerical simulations using a generalized predictor–corrector scheme adapted for variable-order systems, whereas many previous works are limited to theoretical discussion only. These aspects collectively highlight the novelty and originality of the proposed research methods.

### Definition 6.1

^71^ Hyers-Ulam stability provides in the variable order Caputo fractional enzyme kinetics model (2). If there exists non-negative constants, $\psi _{j}$ such that $\epsilon _{j}$ for $j \in 1,2,3,4$ the function that $(\hat{\mathbb {S}}, \hat{\mathbb {E}}, \hat{\mathbb {H}}, \hat{\mathbb {P}})$ satisfies

13 $$\begin{aligned} {\left\{ \begin{array}{ll} \left. \right| D^{\delta (t)} \hat{S}({t})-\mathscr {L}_{1}({t}, \hat{\mathbb {S}})\left| \le \epsilon _{1},\left. \right| D^{\delta (t)} \hat{E}({t})-\mathscr {L}_{2}({t}, \hat{E})\right| \le \epsilon _{2}\\ \left. \right| D^{\delta (t)} \hat{H}({t})-\mathscr {L}_{3}({t}, \hat{\mathbb {H}})\left| \le \epsilon _{3},\left. \right| D^{\delta (t)} \hat{\mathbb {P}}({t})-\mathscr {L}_{4}({t}, \hat{\mathbb {P}})\right| \le \epsilon _{4} \end{array}\right. } \end{aligned}$$

Fulfills the model (2), and exists $(\mathbb {S}, \mathbb {E}, \mathbb {H}, \mathbb {P})$

14 $$\begin{aligned} {\left\{ \begin{array}{ll}\Vert \mathbb {S}-\hat{\mathbb {S}}\Vert \le \psi _{1} \epsilon _{1}, \quad \Vert \mathbb {E}-\hat{\mathbb {E}}\Vert \le \psi _{2} \epsilon _{2}, \\ \Vert \mathbb {H}-\hat{\mathbb {H}}\Vert \le \psi _{3} \epsilon _{3}, \quad \Vert \mathbb {P}-\hat{\mathbb {P}}\Vert \le \psi _{4} \epsilon _{4} . \end{array}\right. } \end{aligned}$$

where $\mathscr {L}_{j}, j \in 1,2,3,4$ are given in (4).

### Remark 6.1

Suppose there exists a continuous function $\vartheta _{1}$ such that $\mathbb {S}$ satisfies the first inequality in (3).

1. $$\left| \vartheta _{1}(s)\right| \le \epsilon$$, and
2. $$D^{\delta (t)} \hat{\mathbb {S}}(s)=\mathscr {L}_{1}(s, \hat{\mathbb {S}}(s))+\vartheta _{1}(s)$$.

### Theorem 6.1

*Assuming the hypothesis* $\mathscr {H},$ *then the model (*2*) is Hyers-Ulam stable if* ${\frac{{{t}}^{\delta (t)}}{ \Gamma (\delta (t)+1)} } \Phi _{j} \le 1,$ *for* $j \in \mathscr {N}_{1}^{4}.$

### Proof

Consider $\epsilon _{1}>0$, and the function $\mathbb {S}$ be arbitrary for that, $\left. \right| D^{\delta (t)} \widehat{\mathbb {S}}({t})-\mathscr {L}_{1}({t}, \widehat{\mathbb {S}}) \mid \le \epsilon _{1}$.

Then it follows as a function $\vartheta _{1}$ with $\left| \vartheta _{1}({t})\right| <\epsilon _{1}$, satisfying $D^{\delta (t)} \hat{\mathbb {S}}({t})=\mathscr {L}_{1}({t}, \hat{\mathbb {S}})+\vartheta _{1}({t})$.

As a result,

$$\begin{aligned} \hat{\mathbb {S}}({t})=\,&\hat{\mathbb {S}}(0)+\frac{1}{\Gamma (\delta (t))} \int _{0}^{t}({t}-v)^{\delta (t)-1} \mathscr {L}_{1}(v, \hat{\mathbb {S}}(v)) dv\\&+\frac{1}{\Gamma (\delta (t))} \int _{0}^{t}({t}-v)^{\delta (t)-1} \vartheta _{1}(v) d v. \end{aligned}$$

Here $\mathbb {S}$ be the variable order Caputo fractional for the enzyme model (2) as a unique solution. Finally, we obtain

$$\begin{aligned} \hat{\mathbb {S}}({t})=\,&\hat{\mathbb {S}}(0)+\frac{1}{\Gamma (\delta (t))} \int _{0}^{t}({t}-v)^{\delta (t)-1} \mathscr {L}_{1}(v, \hat{\mathbb {S}}(v)) d v \end{aligned}$$

$$\begin{aligned}&\text{ as } \text{ so } \text{ far, } \\&\begin{aligned} |\hat{\mathbb {S}}({t})-\mathbb {S}({t})|=&\frac{1}{\Gamma (\delta (t))} \int _{0}^{t}({t}-v)^{\delta (t)-1} |\mathscr {L}_{1}(v, \hat{\mathbb {S}}(v))-\mathscr {L}_{1}(v,{\mathbb {S}}(v))|d v\\&+\frac{1}{\Gamma (\delta (t))} \int _{0}^{t}({t}-v)^{\delta (t)-1} |\vartheta _{1}(v)|d v. \end{aligned} \end{aligned}$$

$$\begin{aligned}&\text{ By } \text{ this } \text{ way, } \Vert \mathbb {S}-\hat{\mathbb {S}}\Vert =\frac{ {\big [\frac{{{t}}^{\delta (t)}}{ \Gamma (\delta (t)+1)}} \epsilon _{1} \big ]}{ {\Big [1-\frac{{{t}}^{\delta (t)}}{ \Gamma (\delta (t)+1)} } \Phi _{1}\Big ]} \end{aligned}$$

Finally, we have

$$\begin{aligned} \psi _{1}:=\frac{{\big [\frac{{{t}}^{\delta (t)}}{ \Gamma (\delta (t)+1)}} \big ]}{ {\Big [1-\frac{{{t}}^{\delta (t)}}{ \Gamma (\delta (t)+1)}} \Phi _{1}\Big ]} \end{aligned}$$

then $\Vert \hat{\mathbb {S}}-\mathbb {S}\Vert \le \psi _{1} \epsilon _{1}$. In the same way, we can obtain the other solutions of

$$\begin{aligned} {\left\{ \begin{array}{ll} \Vert \hat{\mathbb {E}}-\mathbb {E}\Vert \le \psi _{2} \epsilon _{2}, \\ \Vert \hat{\mathbb {H}}-\mathbb {H}\Vert \le \psi _{3} \epsilon _{3}, \\ \Vert \hat{\mathbb {P}}-\mathbb {P}\Vert \le \psi _{4} \epsilon _{4}. \end{array}\right. } \end{aligned}$$

Hence, the variable-order Caputo fractional enzyme model (2) is Hyers-Ulam stable.$\square$

## Numerical simulation

In this section, we utilize the FRK4M to solve the variable order enzyme kinetics model with distinct constant delays. Here, we consider the FRK4M order method for solving this model for $t=200$ and $0<\delta (t)\le 1$ with initial conditions $S(0)=10$, $E(0)=5$, $H(0)=4$, and $P(0)=0.1$. The system parameter values $x=0.0530$ $y=0.012$, $z=0.040$.

**Figure 2:**
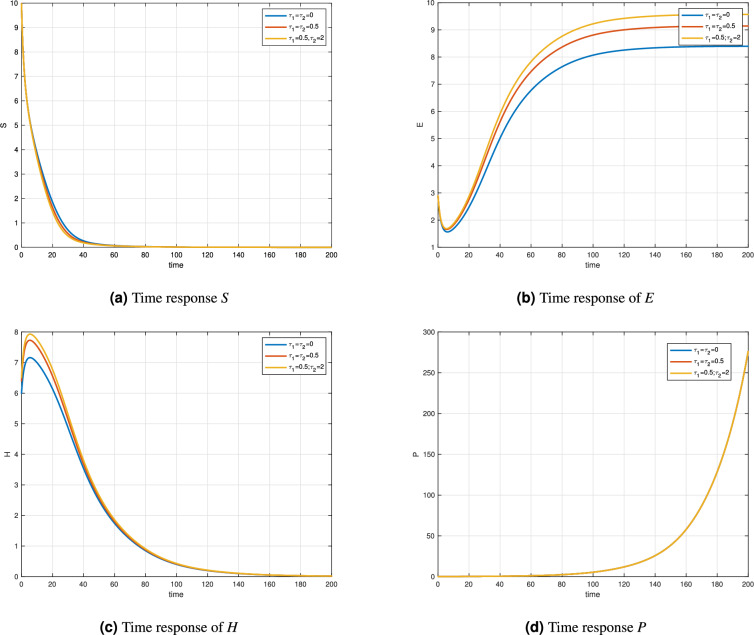
Time responses of Variable order Enzyme model with $\delta (t)=0.98+0.008\cos (t/10)$.

**Figure 3:**
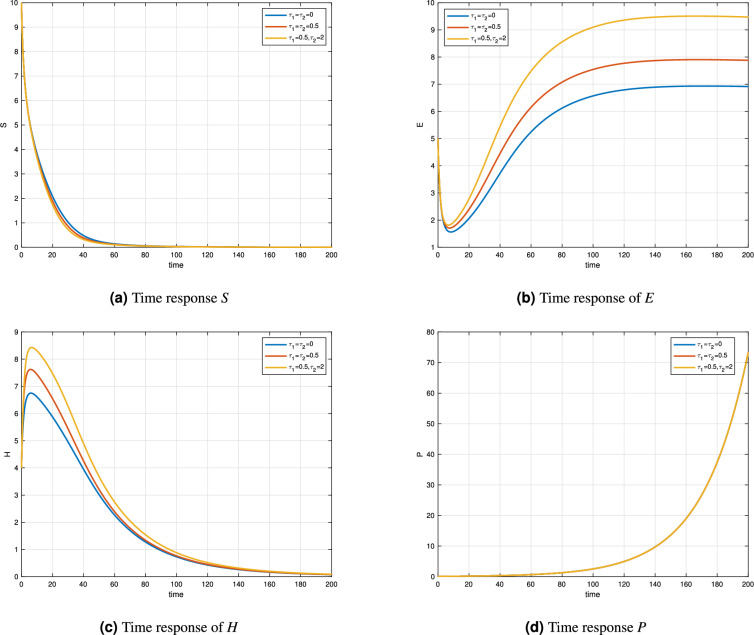
Time responses of distinct variable order Enzyme model with $\delta (t)=0.95+0.001\sin (t/10)$.

Figure 2 and Table 2 illustrates the time responses of a variable-order enzyme kinetic model governed by the fractional-order function $\delta (t)=0.98+0.008\cos (t/10)$. The simulations are conducted under three delay scenarios: $\tau _1=\tau _2=0$, $\tau _1=0.5\ \& \ \tau _2=0$, and $\tau _1=0.5\ \& \ \tau _2=2$. These variations aim to assess the impact of delay on the system’s components: substrate (*S*), enzyme (*E*), enzyme-substrate complex (*H*), and product (*P*). In Fig. 2a, the substrate concentration *S*(*t*) decreases rapidly in all cases, showing typical substrate consumption behavior. The constant delay scenario accelerates this decay slightly, suggesting enhanced enzyme-substrate interactions under memory effects. Figure 2b displays the time evolution of enzyme E(t), which increases over time and eventually stabilizes. Notably, the final concentration is higher under variable and constant delay conditions, indicating more efficient enzyme regeneration when memory is incorporated. Figure 2c shows the enzyme-substrate complex *H*(*t*), which initially peaks and then gradually decays. The magnitude of the peak is more prominent in the presence of delay, especially the time-varying case, implying a stronger initial reaction. Finally, Fig. 2d highlights the product concentration *P*(*t*), which exhibits an exponential increase, especially after $t>100$. The variable delay enhances product accumulation compared to the no-delay case, demonstrating the significant effect of memory on the late-stage dynamics of the reaction. Figure 3 and Table 3 presents the time responses of a distinct variable-order enzyme kinetic model, characterized by a fractional-order function $\delta (t) = 0.95 + 0.001\sin (t/10)$. The figure compares the system behavior under three delay conditions: $\tau _1 = \tau _2 = 0$, $\tau _1 = \tau _2 = 0.5$, and $\tau _1 = 0.5, \tau _2 = 2$. These simulations highlight how delays and memory effects influence the dynamic behavior of substrate (*S*), enzyme (*E*), enzyme-substrate complex (*H*), and product (*P*). Figure 3a, the substrate *S*(*t*) shows a decreasing trend across all cases, reflecting the typical consumption behavior as the enzyme reaction proceeds. The decay is slightly faster under time-dependent delay, which may be attributed to enhanced catalytic interaction due to stronger memory effects. Figure 3b illustrates the time evolution of the enzyme *E*(*t*), which initially dips slightly and then increases towards a saturation point. The response is more pronounced in the variable delay scenario, where enzyme concentration reaches higher steady-state levels compared to the constant or zero-delay cases. Figure 3c shows the enzyme-substrate complex *H*(*t*), which exhibits an initial peak followed by a gradual decline. As seen in previous figures, this behavior is amplified when delay is introduced, especially under time-varying conditions. The heightened peak suggests a more significant initial formation of the complex under memory-influenced kinetics. Finally, Fig. 3d shows the product concentration *P*(*t*), which grows exponentially over time. While all three delay settings yield similar qualitative trends, the time-dependent delay leads to a faster accumulation rate after $t > 150$, demonstrating the effect of fractional memory and delay on long-term product formation.

**Table 2:** Enzyme-substrate complex concentration compartment for $\tau _1=0.5$, $\tau _2=2$ for different orders.

t	$\delta (t)=1$	$\delta (t)=0.97$	$\delta (t)=0.99-(0.001/100)t$
0	4	4	4
20	8.6746	8.0696	8.4733
40	5.2773	5.2181	5.2641
60	2.5465	2.7669	2.6209
80	1.1966	1.4502	1.2788
100	0.5589	0.7726	0.6261
120	0.2604	0.4215	0.3097
140	0.1212	0.2369	0.1557
160	0.0564	0.138	0.0801
180	0.0564	0.138	0.0801
200	0.0122	0.0533	0.0237

**Table 3:** Enzyme-substrate complex concentration compartment for $\tau _1=\tau _2=0.5$ for different orders.

t	$\delta (t)=1$	$\delta (t)=0.97$	$\delta (t)=0.99(0.01/100)t$
0	4	4	4
20	7.5752	7.0755	7.4095
40	4.5005	4.502	4.5078
60	2.1466	2.3824	2.2259
80	0.9959	1.2421	1.0753
100	0.4596	0.659	0.5219
120	0.2116	0.3586	0.2563
140	0.0974	0.2014	0.1281
160	0.0448	0.1174	0.0657
180	0.0448	0.1174	0.0657
200	0.0095	0.0458	0.0195

**Figure 4:**
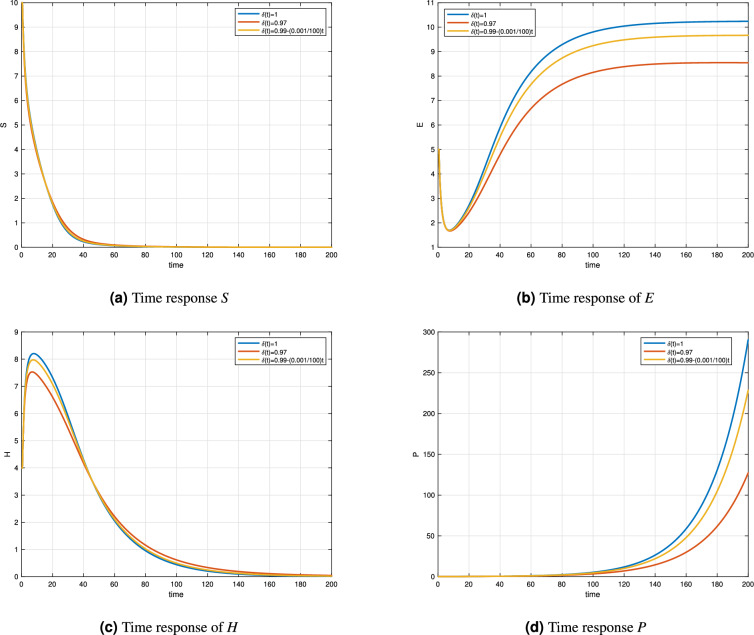
Comparison analysis of integer, fractional and variable order Enzyme model.

Figure 4 and Table 4 illustrates the time-domain behavior of the enzyme system under three scenarios: integer-order ($\delta = 1$), constant fractional-order ($\delta = 0.97$), and variable-order $\delta (t) = 0.99 - 0.001\sin (t/100)$. The plots show the concentration evolution of substrate *S*(*t*), enzyme *E*(*t*), enzyme-substrate complex *H*(*t*), and product *P*(*t*) over the simulation horizon.

As seen in Fig. 4a, the substrate concentration *S*(*t*) decreases over time in all cases due to substrate consumption in the enzymatic reaction. The variable-order model exhibits the fastest decay, indicating a stronger memory effect that accelerates the initial substrate conversion. The fractional-order model lags slightly behind, while the integer-order model maintains the slowest consumption rate.

In Fig. 4b, the enzyme concentration *E*(*t*) increases and stabilizes at a steady state. The integer-order case reaches the highest enzyme level, whereas the fractional and variable-order cases stabilize at lower values due to persistent memory damping. This suggests that non-integer dynamics may better capture enzyme regulation mechanisms that hinder excessive accumulation.

Fig. 4c shows the enzyme-substrate complex *H*(*t*), peaking early before declining. The variable-order response has a slightly higher and sharper peak, indicating a more active initial binding process. This transient behavior highlights the impact of variable memory in amplifying reaction rates during early dynamics.

Finally, Figure 4d demonstrates the product formation *P*(*t*), which grows rapidly in all models. The variable-order model leads to the fastest accumulation, surpassing both fractional and integer cases after $t > 150$. This behavior underscores the enhanced memory-driven reaction kinetics in variable-order systems, which capture long-term accumulation effects more accurately.

**Table 4:** Composed of one molecule compartment for $\tau _1=\tau _2=0$ for different orders.

t	$\delta (t)=1$	$\delta (t)=0.97$	$\delta (t)=0.99(0.01/100)t$
0	4	4	4
20	6.7753	6.3323	6.6285
40	4.1958	4.1811	4.1979
60	2.028	2.2559	2.1049
80	0.9414	1.1798	1.0183
100	0.4339	0.6261	0.4939
120	0.1995	0.3406	0.2422
140	0.0916	0.1912	0.121
160	0.0421	0.1115	0.062
180	0.0421	0.1115	0.062
200	0.0089	0.0434	0.0184

To highlight the effectiveness of the proposed variable-order fractional model, we compare its dynamics with those of the corresponding classical integer-order model (where the order $\delta (t) = 1$). The simulation results show that the fractional model exhibits smoother and more gradual transitions, capturing memory effects that slow down or accelerate the reaction based on historical states. In contrast, the integer-order model responds more abruptly, lacking the capacity to represent fading or adaptive memory. This comparative study underscores the ability of the fractional framework to more accurately reflect the complex and time-dependent behavior of enzymatic processes, providing deeper biological insight.

## Conclusion

In this study, we introduced a variable-order Caputo fractional derivative into an enzyme kinetics model with time delay to better capture memory effects and the nonlocal behavior of enzymatic reactions. The proposed variable-order Caputo fractional model offers a significant advancement over both classical enzyme kinetics and existing fixed-order fractional models. While the classical Michaelis–Menten model assumes constant, memoryless dynamics, and fixed-order fractional models impose a constant memory effect, our approach allows the model memory to adapt dynamically over time. This enables the model to capture complex kinetic phenomena observed in real enzymatic processes, such as delayed product formation and prolonged transient responses, providing a richer and more realistic representation of biochemical reaction dynamics. By employing fixed-point theory, we established the existence and uniqueness of solutions for the proposed model. Furthermore, the stability analysis was conducted using Ulam–Hyers and generalized Ulam–Hyers concepts, confirming the model’s robustness under perturbations. Through numerical simulations, we demonstrated the intricate dynamics of the system and highlighted the significance of incorporating variable-order fractional differentiation and delay terms in refining enzyme kinetics models. The findings of this work contribute to a more accurate and comprehensive representation of biological catalytic processes, providing a valuable framework for further research in enzyme kinetics and related fields. Future research will extend the variable-order fractional enzyme kinetics model to complex reaction networks and multi-enzyme systems, incorporating state-dependent fractional orders to capture adaptive memory effects from environmental conditions. Machine learning integration will enhance parameter estimation and predictive accuracy, while distributed delays will improve biological realism. Experimental validation against real enzyme data and implementation in biotechnology and pharmaceutical processes will demonstrate practical applicability, supported by robust numerical methods and computational optimization for large-scale industrial applications.

## Data Availability

All data generated or analysed during this study are included in this published article.
